# Context Mining of Sedentary Behaviour for Promoting Self-Awareness Using a Smartphone †

**DOI:** 10.3390/s18030874

**Published:** 2018-03-15

**Authors:** Muhammad Fahim, Thar Baker, Asad Masood Khattak, Babar Shah, Saiqa Aleem, Francis Chow

**Affiliations:** 1 Institute of Information Systems, Innopolis University, Innopolis 420500, Russia; 2 Department of Computer Science, Faculty of Engineering and Technology, Liverpool John Moores University, Liverpool L3 3AF, UK; t.baker@ljmu.ac.uk; 3 College of Technological Innovation, Zayed University, Abu Dhabi Campus, Abu Dhabi 144534, UAE; asad.khattak@zu.ac.ae (A.M.K.); Babar.Shah@zu.ac.ae (B.S.); Saiqa.Aleem@zu.ac.ae (S.A.); 4 University College, Zayed University, Dubai 144534, UAE; Francis.Chow@zu.ac.ae

**Keywords:** context recognition, self-management, unhealthy sitting habits

## Abstract

Sedentary behaviour is increasing due to societal changes and is related to prolonged periods of sitting. There is sufficient evidence proving that sedentary behaviour has a negative impact on people’s health and wellness. This paper presents our research findings on how to mine the temporal contexts of sedentary behaviour by utilizing the on-board sensors of a smartphone. We use the accelerometer sensor of the smartphone to recognize user situations (i.e., still or active). If our model confirms that the user context is still, then there is a high probability of being sedentary. Then, we process the environmental sound to recognize the micro-context, such as working on a computer or watching television during leisure time. Our goal is to reduce sedentary behaviour by suggesting preventive interventions to take short breaks during prolonged sitting to be more active. We achieve this goal by providing the visualization to the user, who wants to monitor his/her sedentary behaviour to reduce unhealthy routines for self-management purposes. The main contribution of this paper is two-fold: (i) an initial implementation of the proposed framework supporting real-time context identification; (ii) testing and evaluation of the framework, which suggest that our application is capable of substantially reducing sedentary behaviour and assisting users to be active.

## 1. Introduction

In past decades, sedentary behaviour has appropriately received considerable attention in both developed and developing countries due to societal changes. People spend most of their time in sedentary activities, and their metabolic health is compromised due to low levels of energy expenditure (e.g., while sitting watching television, working on a computer in the workplace, using a cellphone, driving automobiles, playing video/board games, reading books and lying on the couch [1]). To address sedentary behaviour, some initial clarification is required about the terminology. We refer to all sitting activities in different contexts with an energy expenditure of ≤1.5 resting metabolic equivalents (METs) as sedentary behaviours [2]. Hence, a person is considered sedentary if s/he spends a large amount of the day in such activities. Sedentary behaviours are associated with chronic disease [3], physiological and psychological problems [4], cardiovascular disease, diabetes [5] and poor sleep [6]. The most noticeable one is the high risk of being overweight and obesity, which have become serious public health threats worldwide and comprise the second leading cause of preventable death, trailing only tobacco [7]. Therefore, a self-management approach is required to support self-awareness and promote healthy behaviour to reduce the health risks caused by sedentary behaviour. The research community has suggested that new technologies like smartphone alerts of elapsed sedentary time and short breaks during prolonged sitting could be adopted in our daily routines [1,8].

Recently, activity trackers such as Fitbit [9], smartphone apps such as Google Fit [10] and smartwatch activity apps [11] can recognize many user activities. However, these trackers generate a time-series of user activities, but do not make the user aware of the detected unhealthy behaviour. This paper presents our model to detect the sedentary behaviour patterns and create a personal behaviour profile to store collected information. In the future, these profiles may assist practitioners to counsel the users or predict the future based on everyday rhythms of sedentary activity and past sedentary habits. Integrating smartphone technology has great potential to promote healthy behaviours [12]. Users do not have to wear/carry extra gear to monitor and track their daily routine behaviour. The smartphone has various embedded sensors (e.g., accelerometer, audio, WiFi, Global Positioning System (GPS), Bluetooth, gyroscope, magnetometer), high computational power and storage and programmable capabilities, along with wireless communication technologies [13]. Furthermore, it has become an integral part of our daily routines; and one of the best devices to recognize the user’s context.

In order to mine the contexts of sedentary behaviour, there is a need to develop a ubiquitous system that can track the sedentary elapsed time accurately with all its minor routines ranging from office work to watching television during leisure time. Previously, we conducted a pilot study on micro-context recognition [14] and visualizing the user behaviour over the web through the Internet. In this paper, we propose a user-centric smartphone-based approach to recognize the context of sedentary behaviour based on the onboard accelerometers and audio sensors of the smartphone. We compute the acceleration and acoustic features over the collected sensory data streams and mine the contexts by applying the non-parametric nearest neighbour classification algorithm [15]. The main contribution of this paper is two-fold: (i) an initial implementation of the proposed framework supporting real-time context identification; (ii) testing and evaluation of the framework, which prove that our application is capable of substantially reducing sedentary behaviour and assisting humans to be active. The aim of the proposed framework is to monitor human sedentary behaviour in a proactive way. Based on the tracked behaviour, users will be able to monitor and manage their daily routines, which may help them to adopt active lifestyle.

The rest of the paper is structured as follows: Related work and the limitations of existing systems are discussed in Section 2. In Section 3, we provide the proposed architecture and its implementation inside the smartphone environment to track sedentary behaviour. In Section 4, we explain our experimental setup and present the obtained results. We provide a detailed discussion in Section 5 and interventions for our experimental study. Finally, the paper concludes with our findings and proposes future work in Section 6.

## 2. Related Work

Large proportions of the population report insufficient physical activity, high volumes of sedentary behaviour and poor sleep [16,17,18,19,20,21]. The most common methods to capture sedentary behaviour are self-reporting diaries, direct observations, smartphone applications and wearable devices [22]. Self-reporting diaries and direct observation mechanisms are difficult with respect to recording daily routines of a long duration and are time consuming to manage on a daily basis. On the other hand, wearable devices and smartphone applications represent a comprehensive way to monitor sedentary behaviour continuously. We report the efforts of the research community in monitoring sedentary behaviour in the following section.

### 2.1. Wearable Devices

Globally-accepted wearable devices to monitor sedentary behaviour are ActiGraph and actiPAL [23]. Matthews et al. [24] used the ActiGraph device to record the acceleration information and estimate the body movement. The wearable device was set to provide information in 1-min epochs. Participants wear this on their right hip attached with an elastic belt. After the collection of the data, the device was attached to computer, and data were analysed using specially-developed software. This mechanism is intrusive, and the user needs to attach the device to his/her body. Chelsea et al. [25] explored how the DigMem system is used to successfully recognize activity and create temporal memory boxes of human experience, which can be used to monitor sedentary behaviour. Users can track where they exactly were, what they were doing and how their bodies were reacting. Their solution is comprised of multimodal sensors including GPS, camera, ECG monitor, environmental sensor, sound and accelerometer. This notwithstanding, such a system is still not a handy solution to embrace in daily routines in order to promote self-awareness.

Stratton et al. [26] created an intelligent environment to monitor and manipulate the physical activity and sedentary behaviour. They also discussed the broad range of approaches already designed to increase physical activity among different populations. Their proposed solution is obtrusive due to the need to wear an additional sensor to monitor the sedentary behaviour. Such methods are intrusive and unable to process the sensor data inside the devices. Furthermore, they provide limited information about the sedentary behaviour in everyday routines. One of the drawbacks of wearable devices is the inability to detect the contextual information.

### 2.2. Smartphone Applications

Qian et al. [1] explored smartphone usage to predict sedentary behaviours. They were able to classify user contexts such as location, time and application usage to predict if the user would be sedentary in the coming hours. Their methodology is still unable to distinguish between different types of sedentary behaviour. Dantzig et al. [27] developed the SitCoach mobile application to monitor the physical activity and sedentary behaviour of office workers. The objective of their research was to avoid prolonged sitting by providing timely information to the user in terms of alert messages. They concluded that mobile applications can motivate people to take regular breaks from long sitting. Shin et al. [28] developed a mobile application to recognize user sedentary activity using a mobile device. Their method was based on rotated acceleration using quaternions, which classified sedentary behaviour with higher accuracy. However, their application required server-side processing to classify user activities patterns. It is seen that many systems and models were therefore proposed to track sedentary behaviour; however, they had limitations.

Our proposed approach is to expand upon the lessons learned from existing research work and to enable the detection of the contextual information by utilizing the embedded sensors of the smartphone and processing data in real time inside the smartphone environment.

## 3. Methodology

**Figure 1:**
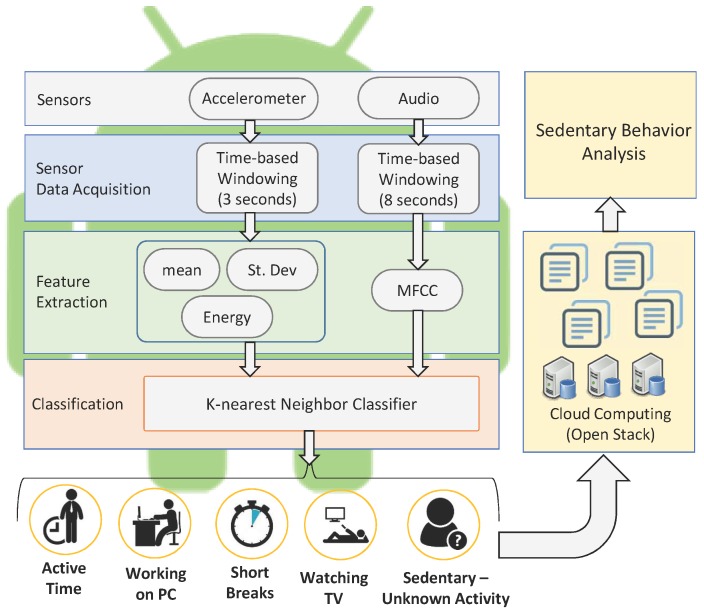
The proposed architecture of context mining. MFCC, Mel-Frequency Cepstral Coefficient.

The contextual mining of sedentary behaviour consists of: (a) the smartphone environment to mine the sensory data streams; (b) cloud computing infrastructure to make it an acceptable and usable solution; and (c) sedentary behaviour analysis. The proposed model is illustrated in Figure 1, and the details of the sub-components are as follows.

### 3.1. The Smartphone Environment

We implement our proposed model using the most competitive open source Google Android platform (Ice Cream Sandwich) [29]. The developed system’s components are detailed as follows.

#### 3.1.1. Sensor Data Acquisition

We collect the temporal sensory data stream of the onboard tri-axial accelerometer and audio sensor of the smartphone. The accelerometer sensor is capable of measuring the acceleration in three orthogonal directions (i.e., *x*, *y* and *z* axis). These raw signals need to be pre-processed to segment the continuous temporal data before extracting the feature set. Therefore, we apply the time-based windowing method to divide it into fixed time segments (i.e., 3 s) [30]. The selection of time-based windowing is based on the its good handling of continuous data [15,28,31]. The audio data stream is an important source to know the user contexts by processing the environmental sound. We collected the audio data stream and applied signal segmentation by dividing it into fixed time segments (i.e., 8 s). The duration of fixed time segments is based on the analysis of audio data, and it will be enough to process for mining contexts. In order to maintain the user privacy, we did not store the audio signal, nor accelerometer signal, but rather, we processed it immediately in real time.

#### 3.1.2. Feature Extraction

**Figure 2:**
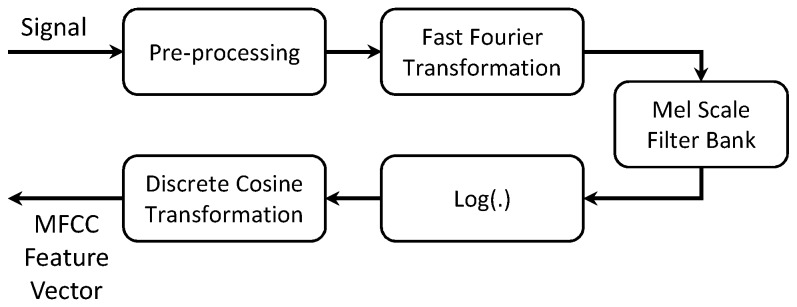
Block diagram of MFCC feature vector calculation.

Feature extraction is the most important part of mining contexts, since the selected features play a crucial role in determining the user’s situation. In the past, many complex features extraction techniques such as Principal Component Analysis (PCA) followed by Linear Discriminant Analysis (LDA) [32] and wavelet features [33] were used; however, they are computationally expensive and difficult to implement inside the smartphone environment, as they require a strong statistical background. Many researchers reported that simple and low cost computational features, such as mean, median and standard deviation, and low and high pass filters are able to achieve high accuracy [33,34]. First, we solve the orientation issue of acceleration data suggested by Mizell [35] and then reduce the complexity of feature computation for mobile devices by extracting the time and frequency domain features, which are the mean, standard deviation and energy feature. We extract the mean to measure the central tendency, the standard deviation to measure the data spread for different activities and the energy feature to find the quantitative characteristics of the data over a defined time period. In order to capture the characteristics of environmental sound, we extract the Mel-Frequency Cepstral Coefficients (MFCC) feature vector. It is calculated on the basis of Fast Fourier Transformation (FFT), which is closest to the human auditory system due to the utilized Mel-scale filter bank and represented as the short-term power spectrum of a sound [36]. The calculation of MFCC can be structured into several steps. Figure 2 shows the block diagram for calculating the MFCC feature.

After the feature extraction step, the feature vector is supplied to the classifier to know the current context of the user.

### 3.2. Classifier

The classifier has the ability to learn the concept during the training phase, mine the situations and assign the context label during the recognition phase in real time. The extracted feature vectors (i.e., Section 3.1.2) are provided to the classifier for the classification. In the first stage, accelerometer data are classified into “active” or “still”. In the second stage, audio data are classified by the classifier. In the audio data classifier, we take into consideration only two contexts, while the rest of the contexts are recognized as “sedentary-unknown context”. The following sections explain the training and testing of the classifier.

#### 3.2.1. Training Phase

**Figure 3:**
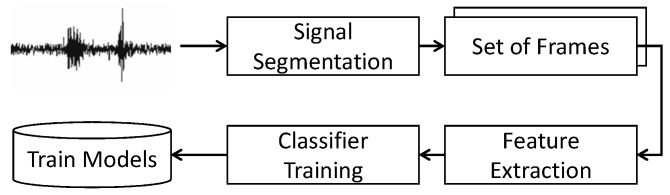
Training of contextual models.

We trained our model over three participants and asked them to annotate the daily routines’ context by miming short duration trials and keeping a note on a piece of paper of the start and end time. We employ non-overlapping time-based windows to cut the signal into equal-length frames. After windowing, feature vectors were extracted (i.e., explained in the feature extraction section) from signal frames and fed into a classifier trainer function to construct a training model. Figure 3 illustrates a block diagram of the training module.

We implement a simple, yet robust non-parametric *k*-nearest neighbour algorithm in our proposed model [37]. It features two stages: the first is the determination of the nearest neighbours, and the second is assigning the context label using those neighbours. In the proposed method, the Euclidean distance metric is applied to find the neighbours, and three neighbours (i.e., *k* = 3) are taken into account. The value of *k* = 3 has been proven to provide good results in some related work and for different settings [30,38,39]. Assume “$C_{fv}$” is the current feature vector that wants to discover the most relevant instances in the context miner “CM”.

(1) $$C_{fv}\leftarrow CM\left(X_{n}\right)$$

where $X_{n}$ is the number of stored training examples to classify the contexts. In order to find the optimal similarities between the current feature vector and selected classifier module, we calculate the Euclidean distance of “$C_{fv}$” with all instances of selected “CM” as follows:

(2) $$Euclidean\;distance:\;d\left(x_{i},x_{j}\right)=\sqrt{\sum_{k=1}^{n}{\left(C_{fv}\left(x_{ik}\right)-CM\left(x_{jk}\right)\right)}^{2}}$$

In Equation (2), the Euclidean distance between two instances “$x_{i}$” and “$x_{j}$” is denoted by “$d_{ij}$”. The distance is calculated for the *k*-th attribute of instance *x*; where, $C_{fv}\left(x_{ik}\right)$ is the feature vector that wants to associate itself with the instances of $CM(x_{jk})$. Based on this structure, most relevant instances are filtered out, and context class labels are assigned by considering the three nearest neighbours. The selection of the *k*-nearest neighbour algorithm is based on one of the most useful and lightweight algorithms for various applications. It is also ranked among the top 10 data-mining algorithms [15].

#### 3.2.2. Recognition Phase

**Figure 4:**
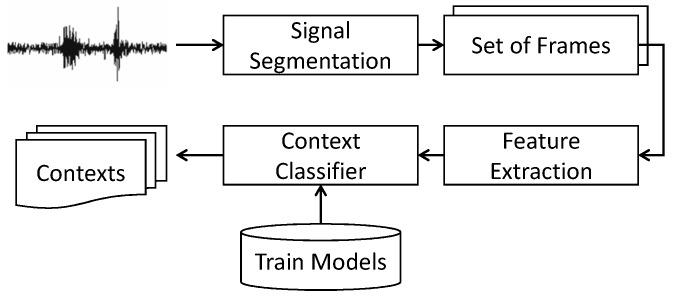
Real-time mining of users’ contexts.

Once we train the model, the trained application will be installed on all participants’ smartphones. Our application is capable of running in the background so that users can use their smartphone for other tasks. Our model is a two-step process, where we process the accelerometer data stream and know the contexts either “still” or “active”. If the classifier labels the context as “still”, we process the environmental sound to recognize the micro-context: working on a PC, watching television, “sedentary-unknown context”. We consider every context as sedentary-unknown if the user is “still” and it does not lie in the defined micro-contexts. Unknown context includes for example sleeping, reading books and attending classes or seminars. In order to preserve privacy, we do not store the environmental sound. We extract the features in real time from the sensory data and fed to the classifier to recognize the micro-contexts. The time scale for inference is set to one-minute epochs, which is sufficient to distinguish among the micro-contexts. If a user is found to be sedentary, then we activate the audio sensor for 8 s to analyse the environmental sound and recognize the micro-context. Furthermore, if we found the micro-context and the user is still in the sedentary state, we check the environment after fifteen minutes to distinguish between the different micro-contexts while staying sedentary. In this way, we save the battery consumption of the smartphone by only checking the environment when the user is in a sedentary state. The training models were used to classify the contexts in real time, as is shown in Figure 4.

**Figure 5:**
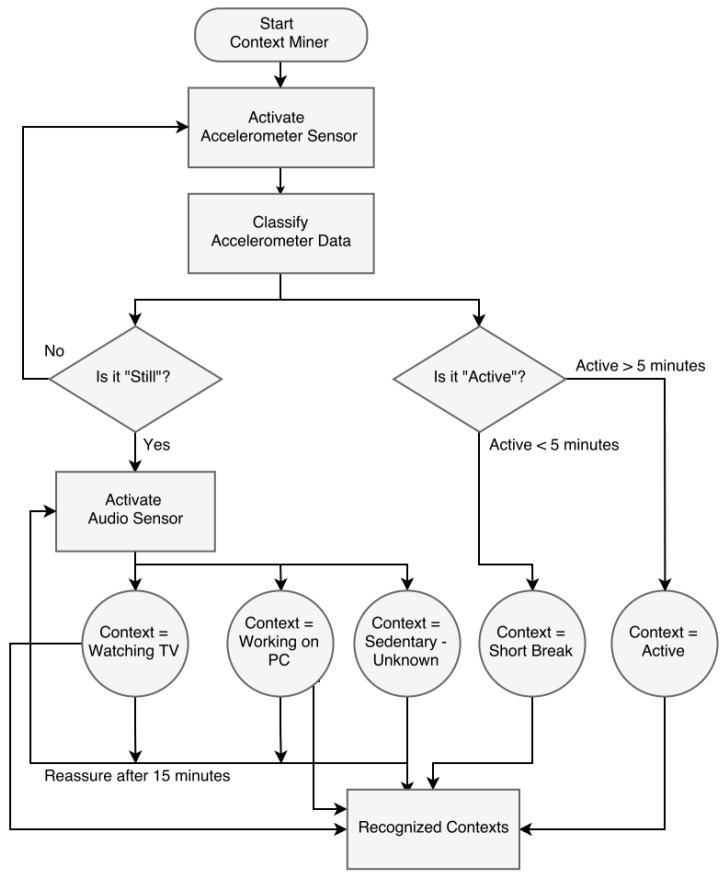
Flowchart of the context miner model.

We processed all these data inside our smartphone application, and furthermore, it requires a ubiquitous service to transfer this contextual information to our private cloud. We deployed the software as a service model, which will automatically scale the services with dynamic provisioning of resources. This approach will reduce the chances of denial of service to the users even at peak usage. It will also enable the user’s phone to be independent in case of any issue with the smartphone. We also present the flowchart of the proposed model in Figure 5.

### 3.3. Cloud Computing

Cloud computing provides scalable and flexible computing model, where resources, such as computing power, storage, network and software, are abstracted and provided as services over the Internet [40] based on a pay-per-use utility model. Cloud computing has been widely used for data hosting and analysis including healthcare/patients’ data storing [41]. It also helps in solving numerous problems in the domain of ambient assisted living. Therefore, we have developed and deployed an open source OpenStack cloud environment in our machine learning research laboratory [42] to provide the user’s profile storage, computing and access services from anywhere at anytime. Our smartphone application recognizes the user’s context in real time inside the smartphone environment and sends the sedentary behaviour profile to our deployed cloud through the Internet. Furthermore, recognized behaviour is analysed to infer the useful information.

### 3.4. Sedentary Behaviour Analysis

Our behaviour analytics provide information about the user daily patterns in terms of sedentary time, active time, short breaks, watching television during leisure time and working in the office while using a computer. These contexts of sedentary behaviour provide better understanding of user’s daily routines and may help users to minimize the amount of prolonged sitting. We are using MPAndroidChart [43] to present information over the smartphone. We create a limited number of credentials that is equal to the number of participants to interact with the system and able to visualize the sedentary behaviour patterns in daily routines.

## 4. Results

**Figure 6:**
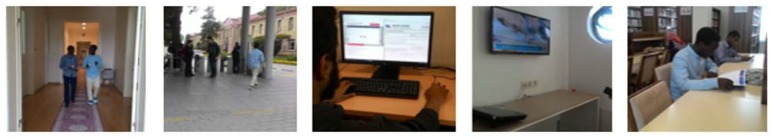
Example photos for “short break”, “active”, “working on a PC”, “watching TV” and “sedentary-context unknown”.

In this section, we evaluate the proposed contextual mining of sedentary behaviour model and present the results of the performed experiments. Our approach belongs to the family of instance-based learning (i.e., *k*-nearest neighbour). Such approaches do not require optimizing the classifier parameters. It stores the training instances and classifies the new data by calculating the similarities of the stored instances. In order to get these training instances, we asked the participants to annotate the daily routines by miming short duration trials and keeping a note of the start and end time of each context. The training dataset is labelled over the time intervals’ information. To assess the performance of our approach, we split the dataset into a ratio of 60:10:30 (i.e., training:validation:test) of the annotated dataset. Our dataset is balanced by considering the equal instances of each considered context. In this experimental setting, the simple performance metric “accuracy” is able to provide correct information about the ability of the model. Initially, we get an overall accuracy of 93% over the collected dataset. We analysed the dataset and performed the data pre-processing. In this step, we discard the first and last few instances of the recorded context. Our analysis showed that start and end instances do not present the true representation of the class. Such a setting enhanced the quality of the training instances in terms of better context representation. In the same experimental setting with the same dataset, we get an accuracy of 98%. The real test setup consists of six volunteer graduate students. The participants installed our developed application on their smartphone for two weeks in order to have enough time to analyse the significance and to perform a comparative analysis of sedentary behaviour. Examples of the scenes of context mining are shown in Figure 6.

**Figure 7:**
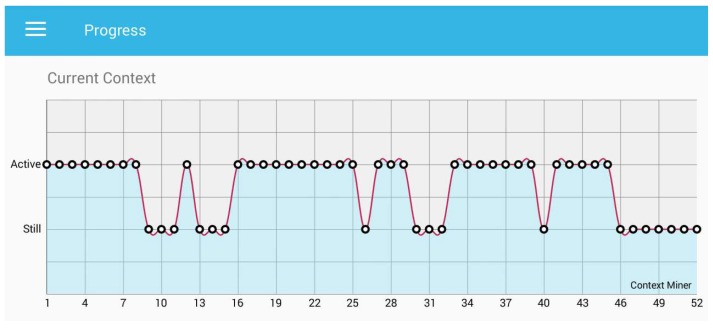
Current progress of the user in the last hour.

**Figure 8:**
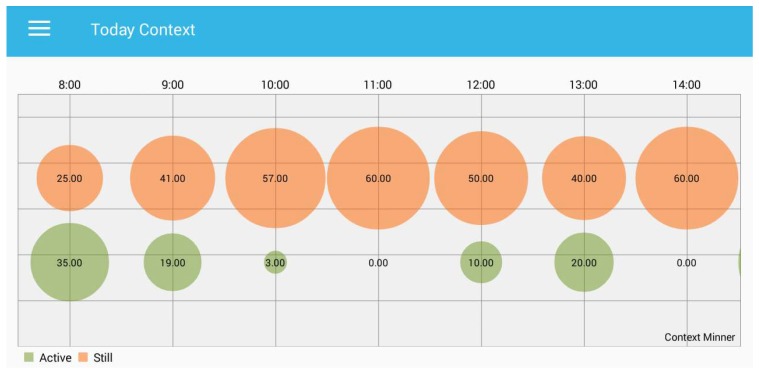
Hourly sedentary behaviour recognition.

In Figure 7, we can observe the progress of the last hour in real time by identifying the context, either active or still. We can see in the “progress graph” (i.e., Figure 7) that the *x*-axis presents the recognized context, the while *y*-axis provides the time stamp in minutes. Furthermore, each point presents each minute of the human behaviour and reports the information about the last 52 min. The annotation of the recognized context shows that the participant was waling from the dormitory to the campus. A user can also visualize the hourly status of the sedentary behaviour of the day. We presented the hourly status of the behaviour, which is the “today context”, as shown in Figure 8.

In Figure 8, each bubble presents the number of minutes, and the size of each bubble increases or decreases with the recognized context. For example, at 11:00, the person is in sedentary activity for the whole 60 min. We can also observe that 0.00 means that person is not active even for a single minute.

**Figure 9:**
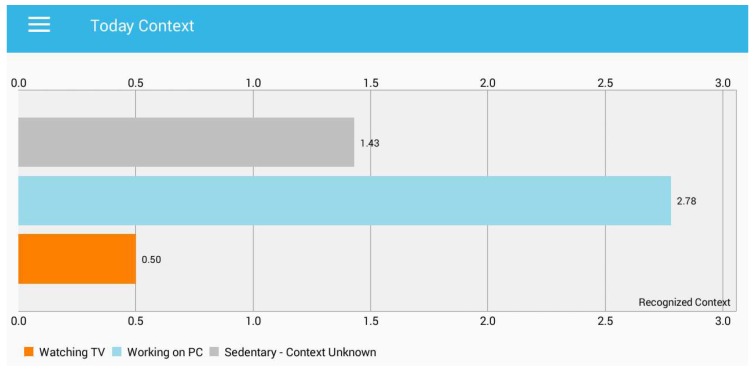
Micro-context recognition.

In order to provide rich contextual information, we facilitate the user awareness about the micro-contexts of sedentary behaviour, which explains how much time the user spent watching TV, working on a PC or sedentary-context unknown. As we discussed earlier, our micro-context recognition list is very limited due to limited processing of environmental sound. Figure 9 shows the details of micro-contexts that our model identifies by processing the environmental sound.

**Figure 10:**
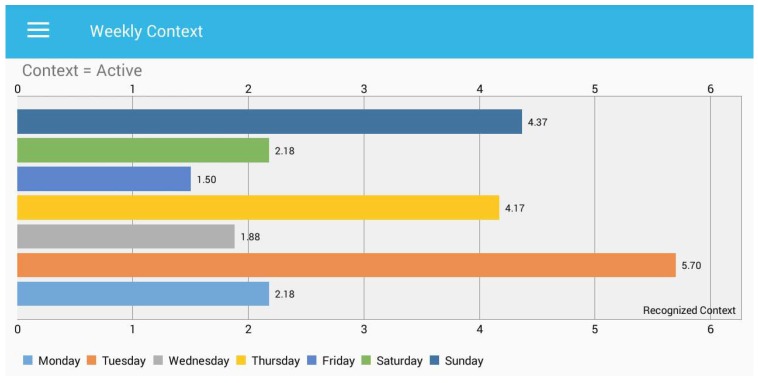
Total time spent during a week while being “active”.

In Figure 9, the *x*-axis represents the time in hours, while *y*-axis presents the recognized micro-context. All the sedentary contexts other than watching TV and working on a PC are considered as sedentary-context unknown. In the unknown context, a user can be located on a public bus, in a library, in a cafeteria, sleeping or any other situation. We also present the entire week of behaviour in terms of recognized context and visualize it through our developed application. The user can query any specific context from the recognized context to get the information about the time spent. Figure 10 shows the total active hours of the user each day throughout the week.

**Figure 11:**
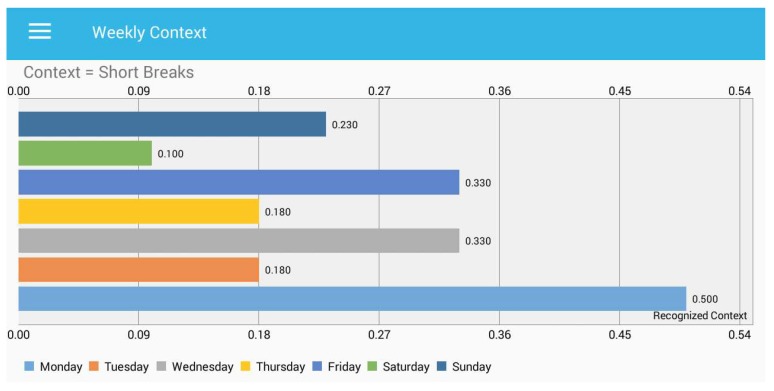
Total time spent during a week for “short breaks”.

It is obvious in Figure 10 that very limited activity is observed during Friday and Wednesday against the context “active”, while the user is very active on Tuesday and Sunday. Figure 11 presents the total duration spent in short breaks for each day. Our model recognized the short breaks between the sedentary hours of a user. Along the *x*-axis, we placed the time in hours, and the *y*-axis presents the days.

**Figure 12:**
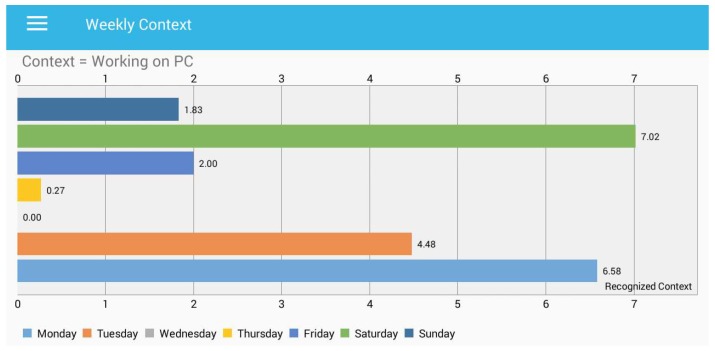
Total time spent during a week for “working on a PC”.

In Figure 11, the user took a small number of short breaks during Saturday, while a large number of short breaks can be seen on Monday. This information about the number of short breaks may help the subject to avoid longer sedentary activity, as well as provide an abstraction to compare different days. In Figure 12, we present the recognized context information while the user is working on a PC. During the wee, the user spent a maximum of 7 h on a PC, while zero hours were recognized on Wednesday.

**Figure 13:**
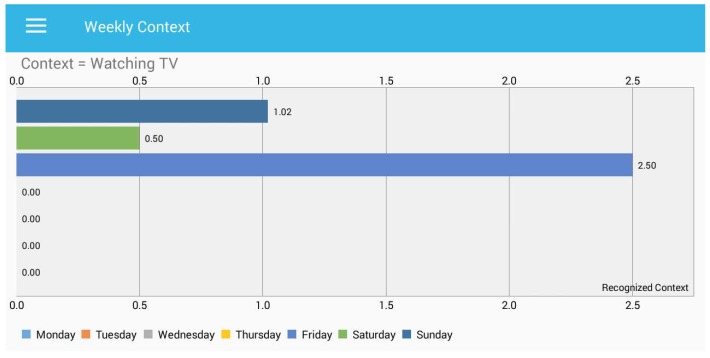
Total time spent during a week for “watching TV”.

In Figure 13, we can observe the total time spent while watching TV during leisure time. In the presented “weekly context”, the *x*-axis presents the context of watching TV, while the *y*-axis presents the time spent in hours. Furthermore, zero means that the user did not watch the television on Monday, Tuesday, Wednesday and Thursday.

**Figure 14:**
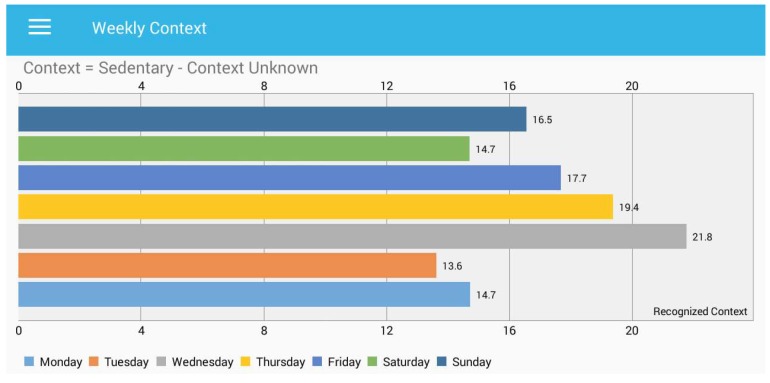
Total time spent during a week for “sedentary-context unknown”.

In Figure 14, we present the sedentary behaviour when the context is unknown. In unknown context sleeping time is also included and sedentary behaviour that is other than watching TV or working on a PC. The *y*-axis presents the context for the whole week, and each bar presents the number of hours spent during the sedentary activity. We found that the SitCoach [27] application is aligned in the same direction of our research. The SitCoach application monitors office workers’ prolonged sitting routines and generates alerts. The alerts may help to reduce sedentary behaviour, and their intervention successfully helps office workers. Their application is restricted in terms of visualization of the user behaviour, as well as mining the micro-contexts. In our proposed research, we are providing rich information to the user about the recognized contexts. We found that self-awareness helps to reduce the sedentary behaviour and motivate the user to avoid prolonging sitting. The following section provides more details about this.

## 5. Discussion

**Figure 15:**
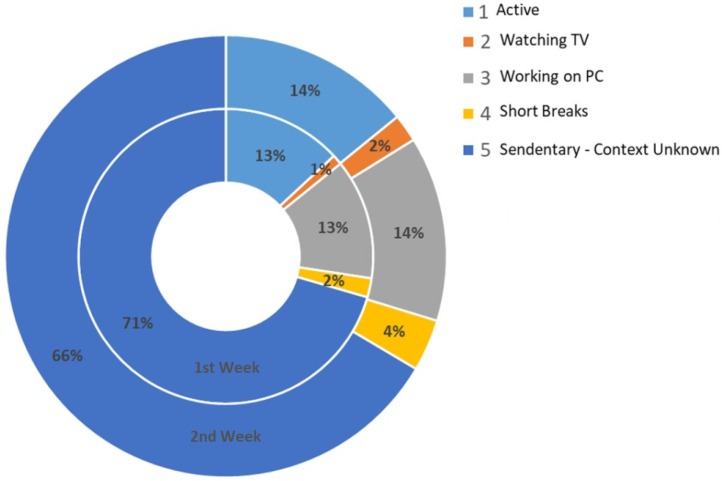
Two-week comparison of the mining contexts.

Context mining of sedentary behaviour and visualization of individual patterns may promote self-awareness to reduce it. In this regard, technology and hand-held smart devices can play a significant role. Whether a person spends much time in sedentary activities is somewhat dependent on the age group, health status, environmental conditions and life roles [12]. Our research goal is to promote self-awareness to reduce sedentary behaviours. Our approach identifies sedentary behaviour based on daily, weekly and monthly patterns. This information can be used to intervene in sedentary behaviour across the working hours, as well as leisure time. Furthermore, it can be used to predict the subject alarming condition while being sedentary in the future. In order to provide rationales for our study, we provide the comparison of one subjects’ two-week comparison of recognized sedentary behaviour. The visualization of recognized contexts is presented in Figure 15.

In Figure 15, the inner circle presents the first week recognized context in percentage, while the outer circle presents the second week contextual information. We can observe quite subtle differences in the recognized sedentary behaviour. For instance, after knowing the sedentary routines, the user took more short breaks during prolonged sitting. In Figure 15, we can observe a 50% increase in short breaks, and sedentary time is reduced from 71% to 66%. The participant also reported that after knowing his/her sedentary behaviour patterns, he/she started making small changes in his/her daily routines. For instance, he/she preferred to use stairs instead of elevators and took short breaks during prolonged sitting.

**Figure 16:**
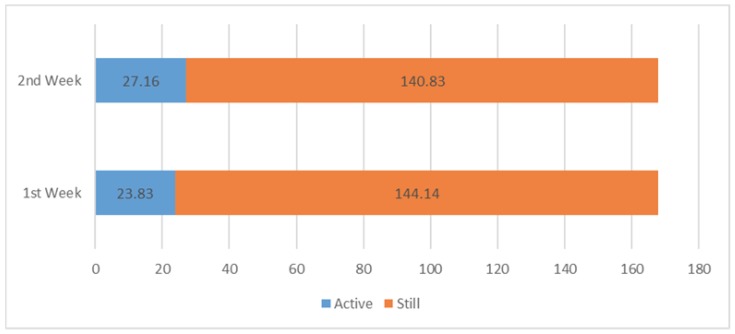
Time spent active or still during two weeks.

In Figure 16, we also present the accumulated results of active or still contexts in terms of hours to get abstract information about the sedentary behaviour.

**Figure 17:**
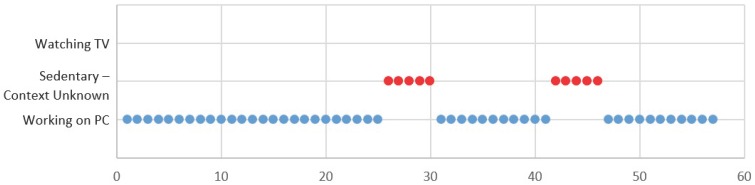
Misrecognized context while working on a PC.

It is obvious from Figure 16 that the participants are becoming more active after knowing the sedentary patterns. Several issues with the study approach were noted throughout. In particular, our trained classifier module classifies the context “sedentary-context unknown” in certain situations where the user is working on a PC and listening to music in the background or using a PC in public places. However, we consider both situations as a sedentary context. It can be seen in Figure 17 that we presented the 57-min contextual data while participants were working on a PC and listening to music. On the other hand, the subject may not have carried his/her smartphone all the time, which may introduce errors in context recognition.

We also collected the participant feedback to find out more about the UX (i.e., user experience). The participants commented that the information they received about the sedentary behaviour is more helpful than they had expected. They found themselves checking the progress and daily status of sedentary behaviour frequently and adjusting their activity accordingly.

## 6. Conclusions

Quantifying sedentary behaviour can provide valuable information about individuals’ daily life patterns. In this research, we proposed a context-mining model to promote self-awareness by monitoring sedentary behaviour and providing a proactive platform for self-management. Participants reported a high level of satisfaction with active or sedentary behaviour, while moderate satisfaction while recognizing the micro-contexts. Micro-context recognition is a complex process that can take place in a wide variety of settings and is influenced by various environmental factors. Furthermore, our model processes the collected sensory data in real time and inside the smartphone environment, which prove the ubiquity of our solution and demonstrate how it does not require any server-side processing, which can obviously undermine privacy. Ultimately, it relaxes the assumption of a strong reliable communication channel to transfer the bulk amount of collected sensory data. The work is ongoing, and we are applying a deep learning model on environmental sound to learn more concrete contexts and situations. We are also working on a dashboard in our application, which will be able to demonstrate the visual representation of users’ progress toward achieving a predetermined standard level of each behaviour for different age groups.
